# Evaluation of *Andrographis paniculata* in a mouse model of influenza A viral infection using oral administration and a translationally relevant dose

**DOI:** 10.3389/fphar.2026.1749384

**Published:** 2026-02-05

**Authors:** Kashif Shamim, Grace C. Burnett, Jin Zhang, Nessma H. Ahmed, Shabana I. Khan, Amar G. Chittiboyina, Ikhlas A. Khan, Gailen D. Marshall, John T. Bates, Nirmal D. Pugh

**Affiliations:** 1 National Center for Natural Products Research, Research Institute of Pharmaceutical Sciences, School of Pharmacy, University of Mississippi, Oxford, MS, United States; 2 Cell and Molecular Biology, University of Mississippi Medical Center, Jackson, MS, United States; 3 Division of Pharmacognosy, Department of BioMolecular Sciences, School of Pharmacy, University of Mississippi, Oxford, MS, United States; 4 Department of Chemistry and Biochemistry, University of Mississippi, Oxford, MS, United States; 5 Department of Medicine, University of Mississippi Medical Center, Jackson, MS, United States; 6 Center for Immunology and Microbial Research, University of Mississippi Medical Center, Jackson, MS, United States

**Keywords:** *Andrographis paniculata*, immune, influenza A (H1N1) virus, mouse model, resilience, translational

## Abstract

**Introduction:**

Ethnobotanically, *Andrographis paniculata* aerial parts have been used to alleviate the symptoms of viral infections, colds, and sore throats. Although the utility of *Andrographis* for these indications has had widespread acceptance, multiple knowledge gaps, regarding mechanisms and specific clinical applications, exist for this botanical. Prior rodent studies have limited applicability to inform future clinical applications due to routes of administration used, nonclinical high doses tested, and exclusive evaluation of chemically purified compounds.

**Methods:**

Experiments used a commercial extract of *A. paniculata* manufactured from aerial parts that was standardized to contain ≥10% andrographolides. *In vitro* evaluation measured NF-κB in THP-1 monocytes, iNOS in RAW 264.7 macrophages and Nrf2 in HepG2 cells. *In vivo* research used a non-lethal influenza A mouse model with two groups (*A. paniculata* treatment and vehicle control). Following infection, mice were gavaged daily for 8 days–treatment group received 250 mg extract/kg body weight (dose equivalent to the human label-recommended daily intake).

**Results:**

The extract inhibited NF-κB and iNOS while activating Nrf2. Although andrographolide contributed to these effects, the findings indicate the presence of additional active constituents that have not yet been characterized. In the mouse model, oral administration of the extract produced a roughly five-fold reduction in lung viral load by day 3 post-infection (*p* < 0.021) compared with control animals.

**Discussion:**

Based on the clinically relevant design of our mouse model, the results lay the groundwork for future preclinical research to determine optimal treatment schedule/dosage and immune mechanisms, thereby informing the design for future resilience efficacy clinical trials.

## 1 Introduction

Ideally, the information gained from scientific research on traditional medicines is translationally relevant to understanding and providing evidence-based guidance on their public health utility in our contemporary society. However, despite significant scientific investigations into these products, there is a growing consensus that the overall data published on their health properties are often inconclusive and even contradictory (Sorkin et al., 2020; Sorkin et al., 2022). To help address these issues, the Office of Dietary Supplements at the United States National Institutes of Health has highlighted the importance of translationally relevant research, rigorous studies and efforts to address critical knowledge gaps in the scientific botanical literature to inform future clinical trials and the public health utility of these products (NIH ODS, 2025).

Our research team has had a long-term focus on investigating botanicals with exceptional clinical potential for improving antiviral resilience by developing the knowledge base necessary to design meaningful future human efficacy studies. A current focus of our program is the investigation of *A. paniculata* (Jayakumar et al., 2013; Jiang et al., 2021). *Andrographis paniculata*, an herbaceous plant from the Acanthaceae family, has been widely used in India, China, and other Southeast Asian countries. In Ayurvedic medicine, it is commonly known as Kalmegh, or the “King of Bitters”, and in Traditional Chinese Medicine as Chuan Xin Lian. The aerial parts of the plant are typically consumed and included in numerous medicinal preparations. Ethnobotanically it is used for its “cold property” to remove heat (fever) and toxins from the body, thereby having purported value in the treatment of viral infections, colds, sore throat, and other health conditions (Jiang et al., 2021). For example, in Thailand, it is included in the National List of Essential Medicines as a first line herbal remedy to reduce fever and sore throat (Kanokkangsadal et al., 2023). Evidence indicates that diterpene lactones contribute to the antiviral and immunomodulatory properties of *A. paniculata* (Jiang et al., 2021). Four major compounds in this class are andrographolide, 14-deoxy-11,12-didehydroandrographolide (DAP), neoandrographolide, and 14-deoxyandrographolide (Pholphana et al., 2016).

The objective of the current research was to evaluate *A. paniculata* using an influenza infection mouse model with factors that aligns with the traditional use of *A. paniculata.* Although three mouse studies have already been published on *A. paniculata*, there are significant knowledge gaps regarding their translational relevance: 1) the oral route of administration was not always used, 2) all doses tested were too high (not aligned with the amount typically consumed by humans), and 3) no studies investigated the effect of ingestion of raw material or crude extracts (only pure compounds were evaluated). The first paper (Ding et al., 2017) reported that andrographolide inhibits influenza A virus-induced inflammation in mice through suppressing nuclear factor kappa B (NF-κB) and Janus kinase-signal transducers and activators of transcription signaling pathways. However, that study was conducted using intraperitoneal administration (not oral) of a pure compound (not an extract or raw plant material). The second study (Cai et al., 2016) reported a protective effect against influenza infection in mice using oral administration of DAP at 500 or 1000 mg/kg/day. These doses are, however, more than 100 times higher than the amount of DAP that would be present in a recommended human daily dose of *A. paniculata–*calculated based on a daily human intake of 3 g of *A. paniculata* (Bone, 2001), DAP present at 0.4% dry weight of plant material (Pholphana et al., 2016), and allometric scaling used to convert mouse to human equivalent dose (Nair and Jacob, 2016). In the third paper (Chen et al., 2009) oral administration of andrographolide at 500 mg/kg/day in a mouse pretreatment model provided a protective effect against three influenza virus strains. This dose is however about 35 times higher than the content of andrographolide within a daily recommended human dose of *A. paniculata*–calculation performed similar to that described above for the second study and assuming that andrographolide is present at 2.3% dry weight of plant material (Pholphana et al., 2016). In the current research we investigated a commercial extract of *A. paniculata* for its antiviral resilience effect by using a nonlethal experimental design reflective of the traditional use of this plant: oral administration of crude extract material and a mouse dosage that is equivalent to the recommended amount traditionally used for human ingestion.

## 2 Materials and methods

### 2.1 *Andrographis paniculata* extracts

A commercial extract of *A. paniculata* (Burm.f.) Nees was purchased from NOW Health Group, Bloomingdale IL (Lot. 3233957, NCNPR voucher code #4635 PR and used for all experiments. The “NOW® Andrographis Extract” was manufactured from aerial plant parts and is a crude alcoholic extract standardized to contain ≥10% andrographolides (each commercial capsule contains 400 mg of the extract). Chemical authentication of the extract material was previously performed by our research team (Avula et al., 2025) and it was determined to contain 43.4 mg of andrographolide (10.9% content) and 55.2 mg of total andrographolides (13.8% content) per capsule. Total andrographolides were defined as the sum of andrographolide, DAP, neoandrographolide, and 14-deoxyandrographolide (Avula et al., 2025).

Botanical reference material for *A. paniculata* aerial parts was obtained from our in-house repository (NCNPR voucher code #9491). Previous analysis of this reference material indicated that it contained 10.7 mg andrographolide and 20.4 mg of total andrographolides per gram of dry plant material (Avula et al., 2025). A crude hydroethanolic extract material was prepared by extraction of powdered plant material with 95% ethanol at 40 °C for 2 h. The liquid extract was collected by filtration and reduced to dryness using a combination of rotavapor, speed vac and freeze-drying (model SAVANT SC 210A, ThermoFisher Scientific, Waltham, MA). The yield of crude extract material was 10.75%.

### 2.2 *In vitro* NF-κB inhibition assay

NF-κB activity was measured using a luciferase reporter gene assay as previously described (Pugh et al., 2001). In brief, THP-1 human monocytes (ATCC, Manassas, VA) were transiently transfected with a NF-κB reporter plasmid and samples were added in the presence and absence of *E. coli* lipopolysaccharide (LPS) serotype 026:B6 (Millipore-Sigma, St. Louis, MO). LPS was added 1 h after sample addition and cells were harvested 4 h later, followed by measurement of luciferase light emission using the ONE-Glo luciferase assay system (Promega, Madison, WI). The Sp-1 reporter plasmid (pGL3-promoter, Promega) was used as a control.

### 2.3 *In vitro* nuclear factor erythroid 2-related factor 2 (Nrf2) activation assay

The assay for Nrf2 activation was performed using a stably transfected NRF2-ARE luciferase reporter HepG2 cell line (Signosis #SL-0046) as described previously (Chae et al., 2025). The cells were seeded in 96-well plates at a density of 30,000 cells/well and incubated for 24 h to reach confluency. The cells were then treated with various concentrations of the test sample (50–200 μg/mL) for 24 h. Curcumin was used as a positive control, while DMSO served as the vehicle control. At the end of the treatment period, the media was removed and the cells were washed with phosphate-buffered saline. Luciferase activity was subsequently measured using Luciferase Assay System (Promega). Luminescence was determined by reading the plate on a Glo-Max plate reader (Promega). The fold increase in Nrf2 activity was calculated by dividing the luminescence of sample treated cells by the luminescence of vehicle treated cells.

### 2.4 *In vitro* inducible nitric oxide synthase (iNOS) inhibition assay

The assay for inhibition of iNOS activity was performed in mouse macrophages (RAW 264.7) induced by LPS as described earlier (Zulfiqar et al., 2017). The cells were seeded at 50,000 cells/well in 96-well plates and treated with various concentrations of test samples (3.125–100 μg/mL) for 30 min prior to the addition of LPS, followed by further incubation for 24 h. At the end of the incubation period, nitric oxide generation in the cell supernatant was measured using the Griess reagent. Plates were read at 540 nm using a SpectraMax M5 plate reader (Molecular Devices, San Jose, CA). The inhibition of nitric oxide generation in sample treated cells is expressed as a percentage relative to maximal activation of cells by LPS treatment alone. IC_50_ values were obtained from concentration response curves. Parthenolide was included as a positive control.

### 2.5 Mouse model of experiment influenza viral infection

**FIGURE 1:**
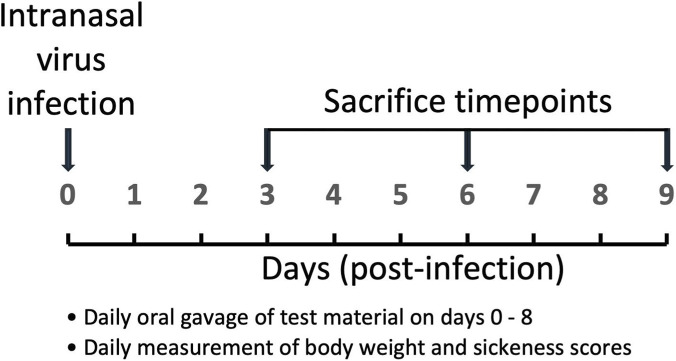
Prodromal anti-influenza treatment model employed for the mouse study investigating *Andrographis paniculata* extract.

A well-established nonlethal mouse model of influenza infection (Cottey et al., 2001) was employed to investigate the *in vivo* activity of *A. paniculata* using a prodromal treatment design (Figure 1; Mir et al., 2024b). A nonlethal experimental design was used to enhance translational relevance since influenza infections are generally nonlethal in the majority of the human population. Male C57BL/6J mice (The Jackson Laboratory, Bar Harbor, ME, 6–8 weeks of age, all purchased at the same time) were used for the study and followed a protocol (2022–1233) approved by the Institutional Animal Care and Use Committee at the University of Mississippi Medical Center. Mice were fed a diet of AIN-93M (Research Diets, Inc., New Brunswick, NJ) for 2 weeks prior to infection and continued throughout the experimental period. Mice were randomized by cage and assigned to two groups (n = 20/group): *A. paniculata* treatment (group 1) or vehicle control (group 2).

All mice, in both groups, were anesthetized and infected via the intranasal route with 15 μL of influenza A/Puerto Rico/8/34 inoculum (2.5 × 10^4^ TCID_50_) on day 0. Viral stocks are maintained in our laboratory that were commercially produced in eggs and sucrose purified (avsbio, catalog #10100374).

Mice were orally gavaged with test material or vehicle control on day 0 (following viral infection) and continued daily through day 8. Group 1 received *A. paniculata* standardized extract dissolved with 5% Kolliphor (Kolliphor® EL, C5135-500G, Millipore-Sigma) in distilled water at a dose of 250 mg/kg body weight. Group 2 received vehicle control. Kolliphor was used to improve solubilization of the extract.

Using a standard allometric conversion factor of mice to humans (Nair and Jacob, 2016), a dose of 250 mg/kg body weight in mice is roughly equivalent to a human dose of 20 mg/kg body weight (250/12.3). For an average adult with a body weight is about 60 kg, this equates to a human daily dose of 1,200 mg of *A. paniculata* extract (20 mg/kg x 60 kg). This aligns with the NOW label-recommended daily dose of 800–1,200 mg (2-3 capsules, each capsule containing 400 mg of extract). Consuming 1,200 mg of extract would expose a human to about 130 mg of andrographolide (43.4 mg/capsule [see section 2.1] x 3 capsules).

Mouse body weights were recorded in grams at approximately the same time each day. Sickness scores were adapted from a *Staphylococcus aureus* challenge model (Blättner et al., 2016) and based on loss of body weight in combination with ruffled fur, hunched posture, reduced motility, labored breathing, irritation around the eyes, and any other observed clinical complications (e.g., paralysis, cold to the touch). Assessors were not blinded to the treatment groups. Some mice were euthanized prior to the intended time point if an animal exhibited >25% weight loss relative to its starting weight and at least two other signs of illness. The remaining mice were euthanized on days 3 (N = 6/group), 6 (N = 5/group), and 9 (N = 4/group).

### 2.6 Quantitation of viral load in lung homogenates

Lungs were harvested from mice at the indicated time points. One lung from each mouse was preserved in RNAlater solution (ThermoFisher Scientific, USA). The remaining lung was weighed, and homogenized with a fastprep96 bead homogenizer (MP Biomedicals, Irvine, CA) in 5 mL of EMEM (ATCC) containing protease inhibitor cocktail (P2714-1BTL, Millipore-Sigma). Samples were centrifuged at 13,000 x g for 10 min at 4 °C, and supernatants were passed through a 0.2 μm filter to remove residual debris. Filtered supernatants were collected into fresh RNase-free tubes and frozen at −80 °C until extraction of viral RNA.

Viral RNA was extracted following our previous protocol (Mir et al., 2024a) using the Genesig® Easy DNA/RNA extraction kit (PrimerDesign Ltd., Eastleigh, UK). The quality and concentration of the extracted RNA were assessed using a NanoDrop spectrophotometer (ThermoFisher Scientific, USA). For all samples, viral quantitation was performed using a standard input of 80 ng of RNA per reaction (from a working stock sample with a concentration of 16 ng/μL).

Quantitative RT-PCR was carried out using the Genesig® Advanced H1N1 influenza RT-qPCR kit (PrimerDesign Ltd., UK; catalog no. R00975, kit version 3), targeting the influenza A matrix protein (M1) gene, together with the Precision® Plus One-Step RT-qPCR master mix (PrimerDesign Ltd., Eastleigh, UK). The RT-PCR was performed on a Bio-Rad CFX Connect 96-well thermal cycler (Bio-Rad, Hercules, CA).

The RT-PCR conditions consisted of reverse transcription at 55 °C for 10 min, followed by enzyme activation at 95 °C for 2 min, and subsequently 45 cycles of denaturation for 10 s at 95 °C and data collection/capturing at 60 °C for 1 min, according to the manufacturer’s instructions.

Absolute quantification of the viral genome was performed using standard curves generated from the kit-provided M1 positive control template, prepared as 10-fold serial dilutions ranging from 2 × 10^5^ to 2 genome copies per µL. The viral genome copy numbers were calculated from the standard curve and are reported as viral genome copies (particles) per µL of RNA input, consistent with the standardized RNA input used across all reactions. An internal RNA extraction control supplied with the kit was included to monitor extraction efficiency and PCR inhibition.

### 2.7 Statistical analysis

The statistical significance of differences in body weight, sickness score and viral load between treated and control mice was determined by two-way ANOVA followed by Bonferroni’s comparison test using Prism 10.4.1 P values less than 0.05 were considered statistically significant.

## 3 Results

### 3.1 *In vitro* characterization of *A. paniculata* by measurement of cellular immune targets (NF-κB, Nrf2 and iNOS)

**FIGURE 2:**
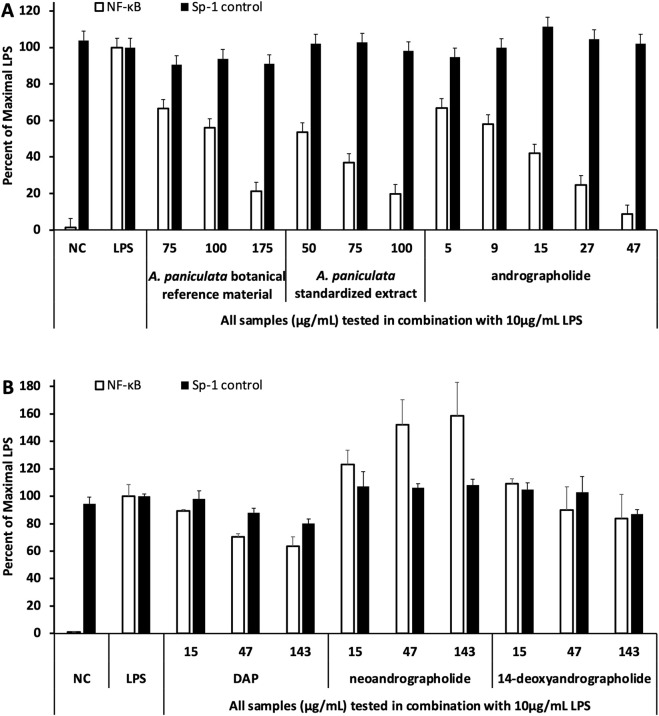
Investigating inhibition of NF-kB directed luciferase expression (in THP-1 cells) exhibited by *Andrographis paniculata* samples (botanical reference material and standardized extract) and andrographolide **(A)** and 3 other andrographolides **(B)**. NC refers to negative control (untreated cells) and the positive control was LPS tested at 10 μg/mL. Sp-1 reporter (pGL3-promoter, Promega) served as the control plasmid. Values represent mean ± standard deviation of duplicate determinations (or triplicate determinations for andrographolide).

Hydroethanolic extract of *A. paniculata* botanical reference material suppressed NF-κB directed luciferase expression in a dose dependent fashion with an IC_50_ value of 112 μg/mL (Figure 2A). The inhibition was selective since no effect was observed on Sp-1 control at any of the tested concentrations. Similar to the botanical reference material, the standardized commercial extract from NOW Health Group also exhibited selective blockage of NF-κB dependent activation of THP-1 cells (IC_50_ value of 55 μg/mL, Figure 2A). Although andrographolide exhibited selective suppression of NF-κB (IC_50_ value of 13 μg/mL), no inhibitory activity was detected for three other phytochemicals belonging to this class of molecules (DAP, neoandrographolide, 14-deoxyandrographolide, Figure 2B). Interestingly, neoandrographolide selectively enhanced LPS-induced NF-κB activation above maximal levels achieved by LPS tested alone.

**FIGURE 3:**
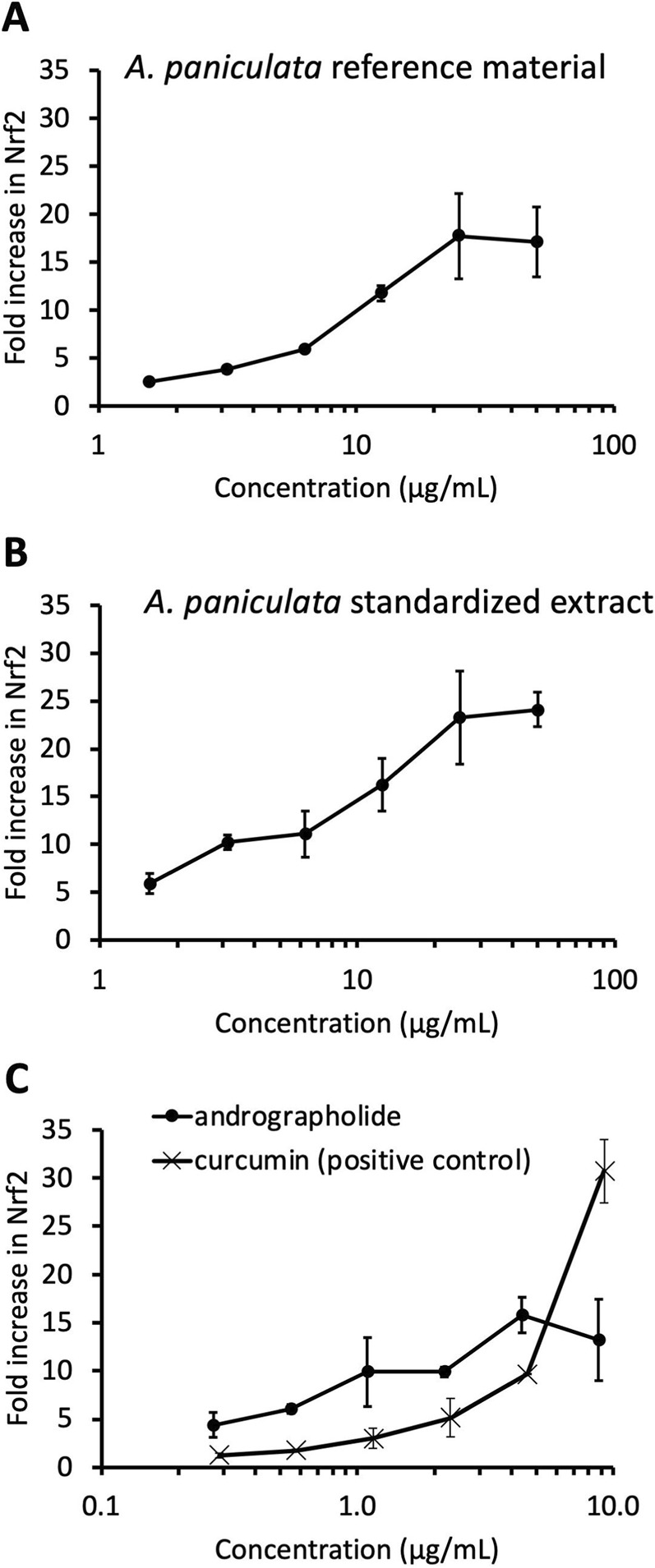
Dose responses of increase in Nrf2 directed luciferase expression (in HepG2 cells) exhibited by *Andrographis paniculata* botanical reference material **(A)**, *Andrographis paniculata* standardized extract **(B)**, andrographolide and positive control curcumin **(C)**. Activation is expressed as fold-induction above control value and values represent mean ± standard deviation of duplicate determinations.

Nrf2-directed luciferase expression was enhanced by 18 ± 4.5‐fold and 17 ± 3.7‐fold at 25 and 50 μg/mL of the hydroethanolic extract of *A. paniculata* botanical reference material, respectively (Figure 3A). Lower doses displayed a concentration dependent enhancement. The NOW® commercial extract also exhibited comparable activity to the reference material (23 ± 4.9-fold and 24 ± 1.8-fold at 25 and 50 μg/mL, respectively, Figure 3B). Andrographolide dose-dependently increased Nrf2 activity, with the highest level observed at 4.4 μg/mL (16 ± 1.8-fold, Figure 3C). At higher doses of andrographolide (8.8 μg/mL), Nrf2 activation appeared to decrease (13 ± 4.7 fold). DAP exhibited weak activation with only 2.2 ± 0.3-fold increase at 8.3 μg/mL (25 µM) but no significant activation was observed at lower concentrations (defined as <2-fold, data not shown). The other two andrographolides (neoandrographolide and 14-deoxyandrographolide) were inactive. As expected, the positive control (curcumin) robustly activated Nrf2 (31 ± 3.3-fold at 9.2 μg/mL, Figure 3C).

**FIGURE 4:**
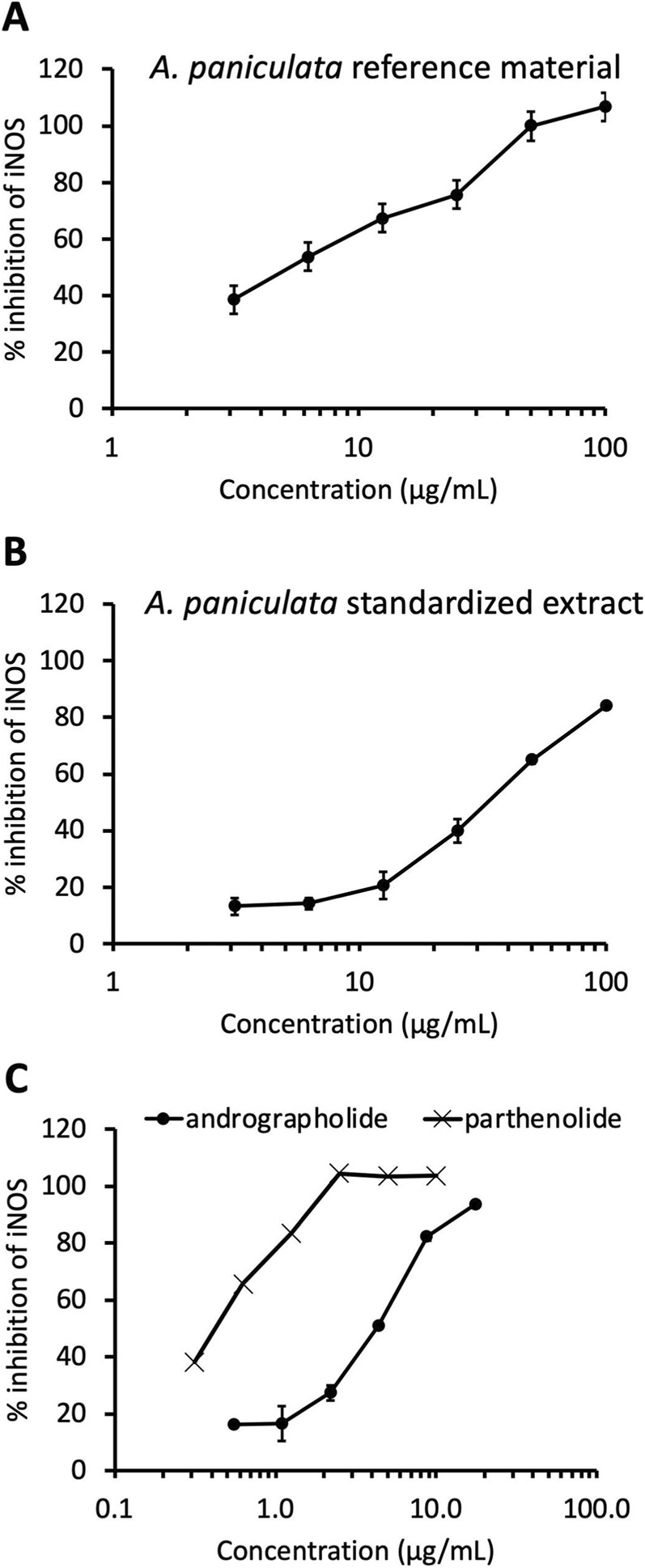
Dose responses of inhibition of iNOS activity (in RAW 264.7 macrophages) exhibited by *Andrographis paniculata* botanical reference material **(A)**, *Andrographis paniculata* standardized extract **(B)**, andrographolide and positive control parthenolide **(C)**. Values represent mean ± standard deviation of duplicate determinations.

Inhibition of iNOS was detected for both the botanical reference material and the NOW® commercial extract with IC_50_ values of 4.9 μg/mL and 32 μg/mL, respectively (Figures 4A,B). Andrographolide also exhibited a dose-dependent inhibition of iNOS with an IC_50_ of 4.4 μg/mL (Figure 4C). None of the other three andrographolides showed any inhibitory effect on iNOS (data not shown). Parthenolide was used as the positive control and demonstrated a potent inhibition of iNOS with an IC_50_ of 0.4 μg/mL (Figure 4C).

### 3.2 Evaluation of *A. paniculata* commercial extract in a mouse influenza infection model on resilience endpoints (body weight and clinical scores) and lung viral load

**FIGURE 5:**
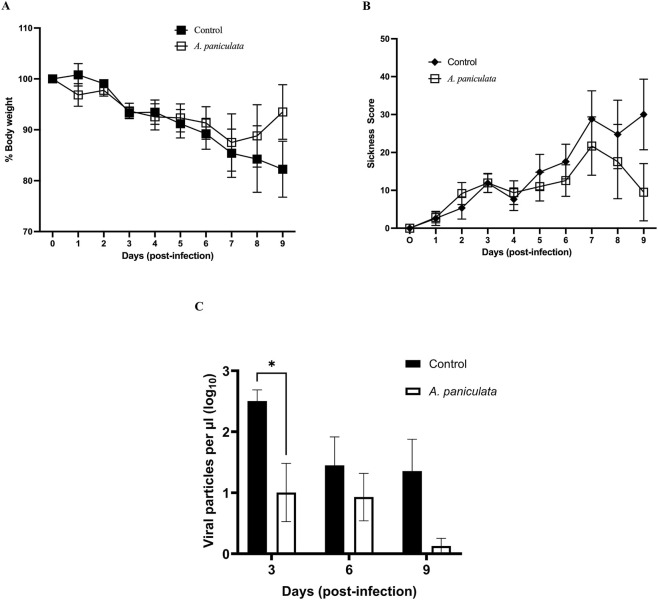
Evaluation of *Andrographis paniculata* standardized extract using a mouse model of viral infection. All mice were challenged with influenza A/Puerto Rico/8/34 (2.5 × 10^4^ TCID_50_) on day 0. *Andrographis paniculata* extract (250 mg/kg body weight) or vehicle control (distilled water containing 5% Kolliphor) was orally administered daily from day 0–8. Each day mean body weight **(A)** and mean sickness scores **(B)** were determined, and values include mice that were euthanized prior to the target time points (due to severity of their symptoms). Lung homogenates were evaluated for viral load on days 3, 6 and 9 post-infection **(C)**. Values on all graphs represent mean ± SEM, *p = 0.021.

Body weight and sickness scores were used as resilience endpoints against infection to represent the ability of the mouse host system to recover or manage the viral challenge. Mice treated with *A. paniculata* experienced less weight loss over the course of infection compared to control mice, though the difference failed to achieve statistical significance (Figure 5A for data presented as means ± SEM and Supplementary Figure S1 for values plotted as individual data points with means ± 95% confidence intervals). Throughout the post-infection period, *A. paniculata-*treated mice maintained body weight more effectively, while the control mice exhibited notable weight loss during days 6–9. The greatest weight loss in control mice was observed on days 8 and 9, with mean losses of 15.8% ± 13% and 17.8% ± 11%, respectively.

For sickness scores, *A. paniculata-*treated mice demonstrate resistance against the symptoms of viral infection (minimal increase in physical signs of illness throughout the post-infection period, Figure 5B for data presented as means ± SEM and Supplementary Figure S2 for values plotted as individual data points with means ± 95% confidence intervals). Although no statistically significant differences were observed in the control mice, sickness score increased between days 6–9, with maximal increases on days 7–9 (27 ± 19, 25 ± 18, and 30 ± 19, mean values ± SEM respectively, Figure 5B).

The effectiveness of *A. paniculata* as an antiviral botanical was investigated by measuring viral particles in lung tissue. Mice treated with the NOW® commercial extract exhibited a significant reduction in pulmonary viral load on day 3 post-infection compared to the infected control mice (decrease of 81.29% ± 27.88%, *p* = 0.021, two-way ANOVA followed by Bonferroni’s comparison test). Although viral loads were also reduced in treated mice on day 6 and 9, the differences were not statistically significant compared to the control group (Figure 5C for data presented as means ± SEM and Supplementary Figure S3 for values plotted as individual data points with means ± 95% confidence intervals).

## 4 Discussion

Resilience is defined as an individual’s capacity to prevent, adapt and recover from illness or adversity, maintaining or restoring normal functioning. This paradigm can be an extremely useful research tool that could provide a more comprehensive approach to investigating the potential medicinal value of some botanicals. With respect to viral illnesses, disease resilience incorporates the contribution of various host physiological and psychological systems to prevent and/or mitigate the viral challenge by modulating protective pathways such as innate and virus-specific adaptive immune responses. Published clinical research indicates that *A. paniculata* can have both direct antiviral properties as well as immunomodulatory effects. For example, a meta-analysis of 33 randomized controlled trials of *A. paniculata* used to treat acute upper respiratory tract viral infections demonstrated a highly significant clinical effect of mitigating symptom intensity and duration compared to placebo and usual supportive care (Hu et al., 2017). In addition, an open-label study reported that oral administration of *A. paniculata* extract in healthy adults resulted in changes in immune cell populations and cytokine profile (Rajanna et al., 2021). Collectively, these clinical studies support the importance of further investigating *A. paniculata* more comprehensively as an antiviral immune resilience promoter (and not only as a direct acting antiviral agent).

Three cellular targets (NF-κB, Nrf2 and iNOS) were selected for our *in vitro* assessment of *A. paniculata.* NF-κB was chosen since its activation is common in viral infections, and it modulates aspects of the immune system involved with resistance to infection (Ludwig and Planz, 2008). Nrf2, a well-known regulator of oxidative stress, is also a regulator of antiviral interferon (Herengt et al., 2021). The third target, iNOS, has a complex regulatory role in viral infection with potential beneficial and harmful roles. Since nitric oxide can stimulate immune pathways, enhanced level can exacerbate pathology (Perrone et al., 2013) during the excessive inflammatory stage of viral infection, commonly referred to as the “cytokine storm”. Thus, inhibiting the intracellular generation of nitric oxide seems to be a plausible approach to reduce inflammation under such conditions.

In agreement with previous research (Yao et al., 2021), our data demonstrate significant inhibition of NF-κB activity by *A. paniculata*. Andrographolide showed an IC_50_ value of 13 μg/mL, approximately 4.3 times more potent than the commercial extract (IC_50_ value of 55.5 μg/mL, Figure 2A). If all the extract activity is due to andrographolide, then the predicted IC_50_ value for this compound should be 6.0 μg/mL [55.5 μg/mL x 0.109, calculation based on the extract having a 10.9% yield of andrographolide (Avula et al., 2025)]. However, since the experimental IC_50_ of andrographolide was 2.2 times higher than this predicted value (13 μg/mL/6.0 μg/mL), we estimate that andrographolide accounts for about 45% of the extract’s NF-κB inhibitory activity (100%/2.2). This suggests that additional immunologically active chemical components are present within the *A. paniculata* extract.

A similar conclusion that *A. paniculata* contains additional bioactive constituents was also derived from the Nrf2 data. Maximal Nrf2 activation was achieved at 50 μg/mL for the commercial extract (24-fold increase over control), whereas andrographolide peaked at a 16-fold induction at 4.4 μg/mL. The activation of Nrf2 by the commercial extract was 1.5 times higher than the maximal level achieved by andrographolide, implying the presence of additional unidentified Nrf2 inducers in *A. paniculata*.

Inhibition of nitric oxide production in LPS-induced macrophages has been previously investigated for 95% ethanol extracts of *A. paniculata* and the detected active components reside in a crude fraction that contains andrographolide (Siridechakorn et al., 2023). Consistent with this prior study, our results show that andrographolide is an iNOS inhibitor. If all the activity we detected in the commercial *A. paniculata* extract (IC_50_ value of 32 μg/mL) was due to andrographolide, then this compound would have had a predicted IC_50_ value of 3.5 μg/mL (32 μg/mL x 0.109). Since the observed IC_50_ value for andrographolide is close to this predicted value (4.4 μg/mL), it is reasonable to speculate that most of the commercial *A. paniculata* extract activity is due to andrographolide. However, the hydroethanolic extract of the botanical reference material exhibited an IC_50_ value of 4.9 μg/mL (comparable to that of andrographolide), suggesting that additional bioactive compounds are likely present in this sample. We hypothesize that the inhibition of iNOS by *A. paniculata* results from the combination of andrographolide and other compounds, the levels of which vary between samples and independently of each other.

To enhance clinical relevance of our mouse model, three design elements were incorporated into the current research paradigm. First, the investigational product was administered orally, consistent with traditional and current human use of *A. paniculata*. Second, the daily dose was 250 mg/kg body weight, which is calculated (by allometric scaling) to be the human label-recommended dose suggested by the commercial producer of the *A. paniculata* extract tested (NOW Health Group). Third, a crude extract (not a pure chemically defined compound) was used, reflecting the traditional medicinal use of this botanical. Using these three elements, *A. paniculata* was investigated using an influenza experimental infection mouse model that employed a prodromal design–administering the investigational product immediately after infection (during the asymptomatic period). Compared to controls, *A. paniculata* fed mice had significantly decreased lung viral RNA levels, indicating this botanical may have value as an antiviral supplement. During days 6–9 post-infection, the mean sickness scores trended lower with less percent body weight that was lost–although neither parameter was significantly different. A limitation of the current study is its exploratory nature. Future studies will incorporate the following: 1) a power analysis to guide determination of sample size and allow for a more definitive evaluation of the outcomes, 2) analysis of lung histopathology, 3) pharmacokinetic studies once all bioactive compounds have been identified, and 4) evaluation of different doses and determination of optimal timing for start of extract administration–before (a prophylaxis model), after the onset of clinical symptoms (a therapeutic model), or at the time (a prodromal model) of viral infection.

Further work with *A. paniculata* extract is continuing in our group. Based upon preclinical data from our established mouse influenza model, we are currently completing a large clinical pharmacodynamic study using Immulina®, a commercially available extract from *Limnospira* (formally *Arthrospira*) *fusiformis*. These studies focus on a pharmacodynamic design in young (18–59) and elderly (>65) cohorts to establish the kinetics of the developing response, the maximum intensity at various dosages as well as the durability once the agent is discontinued. We are studying these *in vivo* effects of Immulina® on biomarkers for changes in antiviral biomarker profiles such as α and γ interferon production, natural killer cell numbers and activity, anti‐influenza antibody titers and anti influenza cytotoxic T cell activity to establish whether it could be given before (prevention) and immediately after (prodromal) infection as well as after clinical manifestation of infection (therapeutic). Data derived from these experiments will inform development of clinical trials that can evaluate the true potential for Immulina® extract as a preventative, prophylaxis or therapy for viral infections such as influenza. Similar studies are planned for *A. paniculata.* The exact form, dosage and planned duration of these future pharmacodynamic studies will be significantly informed by further preclinical studies with *A. paniculata* as described above.

The model we are using in our current research program centers around influenza. This has current pragmatic clinical potential since Immulina® has a strong safety profile and influenza is still potentially a major cause of morbidity and mortality in susceptible populations globally in the enduring endemic. Availability of botanical-based agents that could well include *A. paniculata* extracts are relatively inexpensive, widely available and generally regarded as safe. This approach to improving/maintaining antiviral resilience has significant public health potential if/when the next viral pandemic occurs.

## 5 Conclusion

In conclusion, the *in vitro* data for the cellular targets support a working hypothesis that the immunomodulatory activities of *A. paniculata* are attributable, at least in part, to the combination of andrographolide and other as yet unidentified phytochemicals. It is unlikely that other predominant andrographolides (DAP, neoandrographolide, 14-deoxyandrographolide) contribute to the crude extract activity since they were found, in purified form, to exhibit minimal or no activity in any of the assays. This does not completely eliminate the possibilities that their activity is dependent on other compounds found in the crude extract. This possibility needs further investigation. The current mouse study achieves our original objective to provide preclinical proof of concept utilizing an established influenza infection model that aligns with the traditional use of *A. paniculata*. The results provide the basis for conducting future preclinical research and next steps would include determination of dose response, optimal treatment schedule, innate/adaptive immune mechanisms, identification of additional bioactive compounds followed by pharmacokinetic studies, and lung histopathology. Data from these additional studies would guide the design and interpretation future clinical trials.

## Data Availability

The raw data supporting the conclusions of this article will be made available by the authors, without undue reservation.
