# Supplementary material for: Evaluation of Andrographis paniculata in a mouse model of influenza A viral infection using oral administration and a translationally relevant dose
Source: Front Pharmacol. 2026 Feb 5;17:1749384. doi: 10.3389/fphar.2026.1749384 (PMC12916569; doi:10.3389/fphar.2026.1749384)
Supplement: Supplementary file 1 [file Supplementaryfile1.docx]

**Supporting Information**

Title of the manuscript: Evaluation of *Andrographis paniculata* in a mouse model of influenza A viral infection using oral administration and a translationally relevant dose

Author list: Kashif Shamim, Grace C. Burnett, Jin Zhang, Nessma H. Ahmed, Shabana I. Khan, Amar G. Chittiboyina, Ikhlas A. Khan, Gailen D. Marshall, John T. Bates, Nirmal D. Pugh


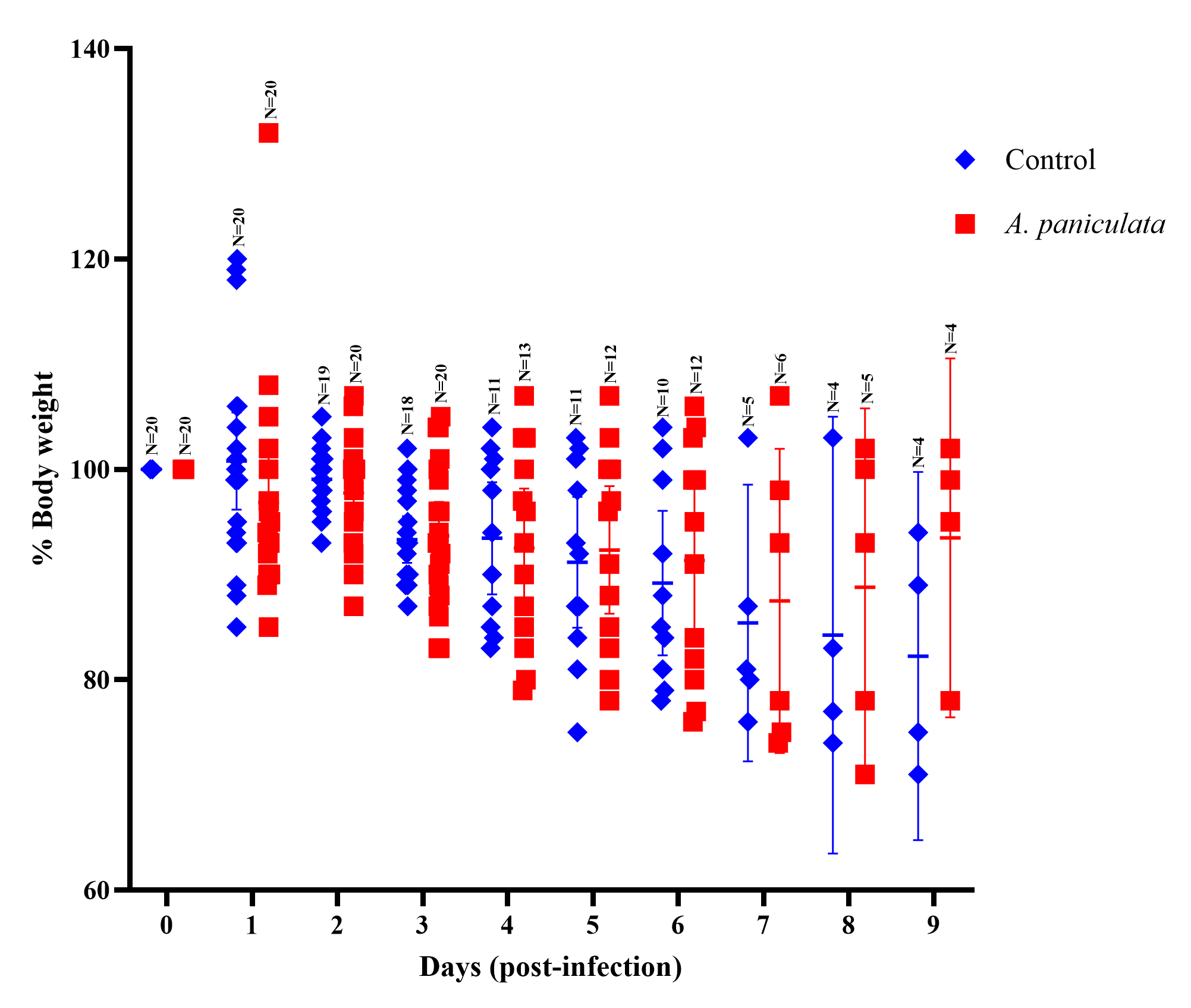


**Fig. S1.** Evaluation of *A. paniculata* standardized extract using a mouse model of viral infection. All mice were challenged with influenza A/Puerto Rico/8/34 (2.5 x 10^4^ TCID_50_) on day 0. *A. paniculata* extract (250mg/kg body weight) or vehicle control (distilled water containing 5% Kolliphor) was orally administered daily from day 0 – 8. Percentage body weight change plotted as individual data points with mean ± 95% confidence intervals, for all mice at each time point. Statistical analysis was performed using two-way ANOVA, and group differences were assessed using Bonferroni’s post hoc test in GraphPad Prism version 10.4.1. No statistically significant differences were observed at any time point. ns = p > 0.05. “N” indicates the number of mice at each time point.


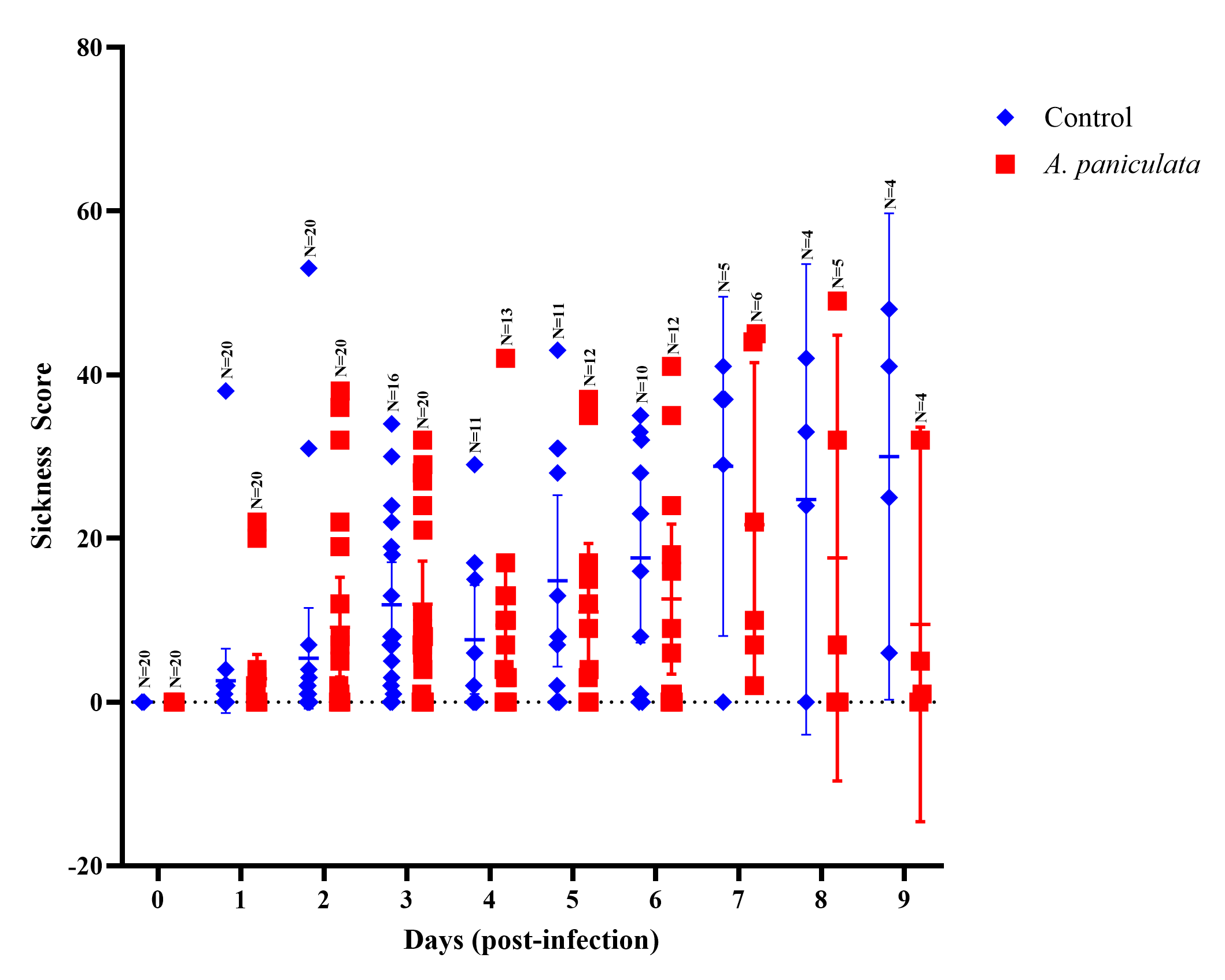


**Fig. S2.** Evaluation of *A. paniculata* standardized extract using a mouse model of viral infection. All mice were challenged with influenza A/Puerto Rico/8/34 (2.5 x 10^4^ TCID_50_) on day 0. *A. paniculata* extract (250mg/kg body weight) or vehicle control (distilled water containing 5% Kolliphor) was orally administered daily from day 0 – 8. Sickness scores plotted as individual data points with mean ± 95% confidence intervals, for all mice at each time point. Statistical analysis was performed using two-way ANOVA, and group differences were assessed using Bonferroni’s post hoc test in GraphPad Prism version 10.4.1. No statistically significant differences were observed at any time point. ns = p > 0.05. “N” indicates the number of mice at each time point.


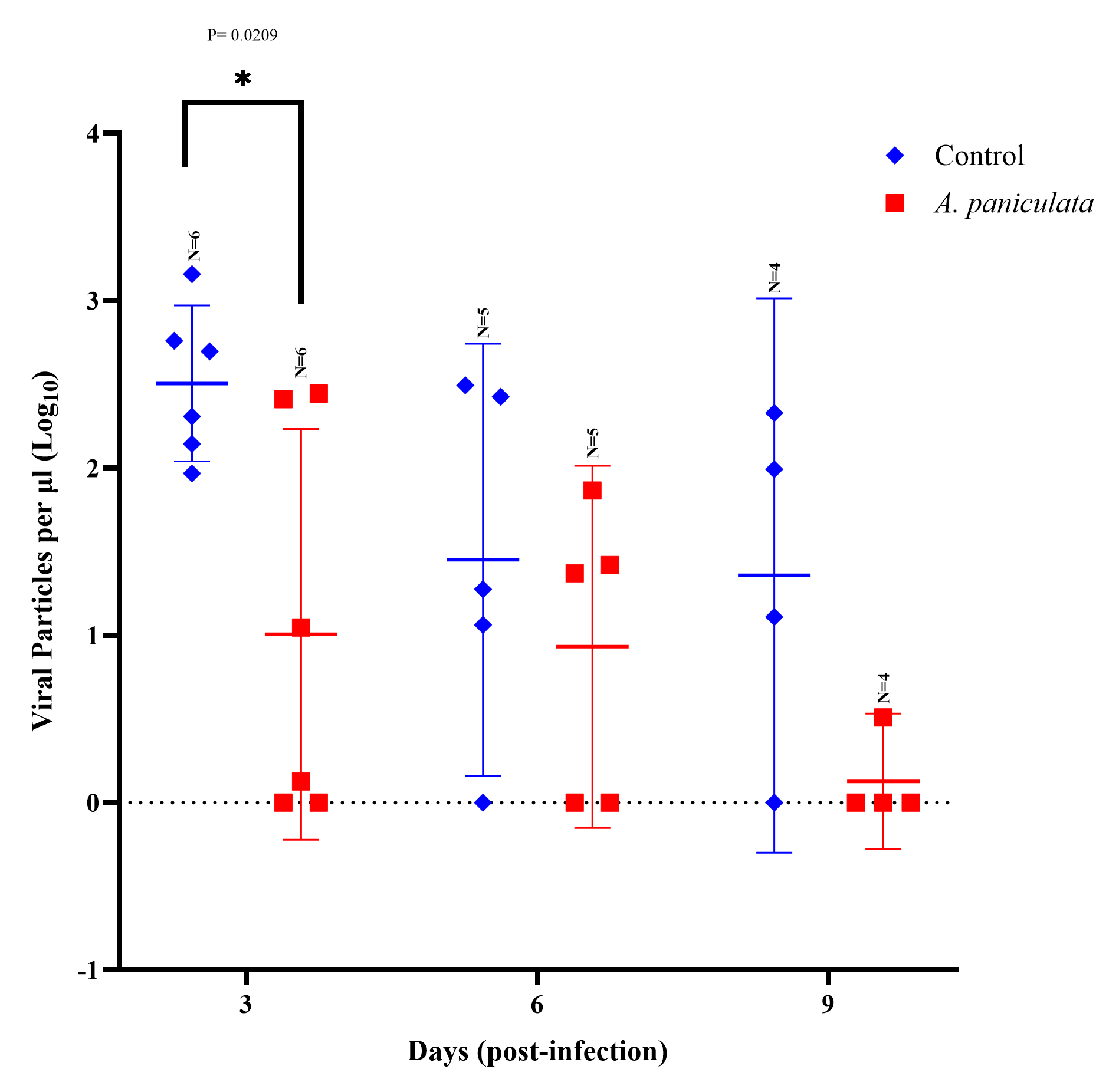


**Fig. S3.** Evaluation of *A. paniculata* standardized extract using a mouse model of viral infection. All mice were challenged with influenza A/Puerto Rico/8/34 (2.5 x 10^4^ TCID_50_) on day 0. *A. paniculata* extract (250mg/kg body weight) or vehicle control (distilled water containing 5% Kolliphor) was orally administered daily from day 0 – 8. Viral load in lung homogenates measured on days 3, 6, and 9 post-infection plotted as individual data points with mean ± 95% confidence intervals. Statistical analysis was performed using two-way ANOVA followed by Bonferroni’s post hoc test in GraphPad Prism version 10.4.1. One time point showed a statistically significant difference (p = 0.0209); all other comparisons were not significant (ns = p > 0.05). All comparisons were made relative to the vehicle control group. “N” indicates the number of mice at each time point.
